# Supplementary figures and images for: Foxp3 depends on Ikaros for control of regulatory T cell gene expression and function
Source: eLife. 2024 Apr 24;12:RP91392. doi: 10.7554/eLife.91392 (PMC11042806; doi:10.7554/eLife.91392)

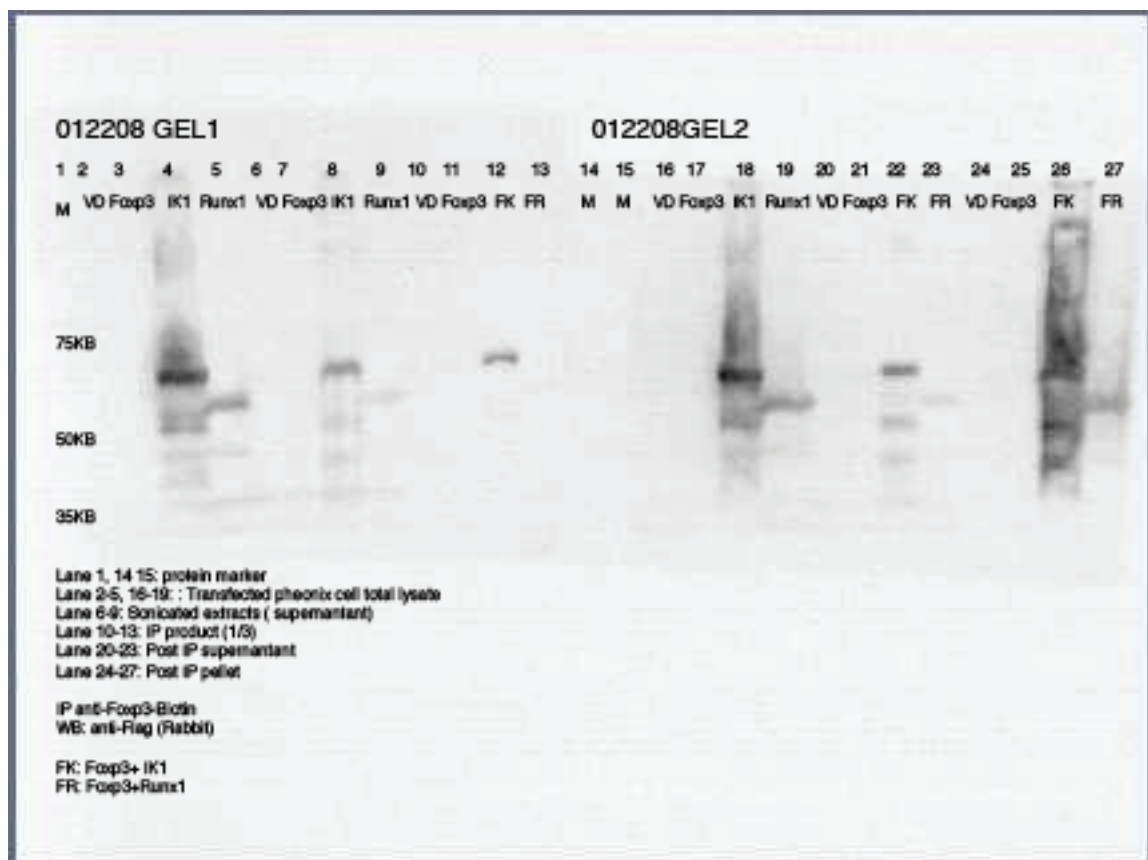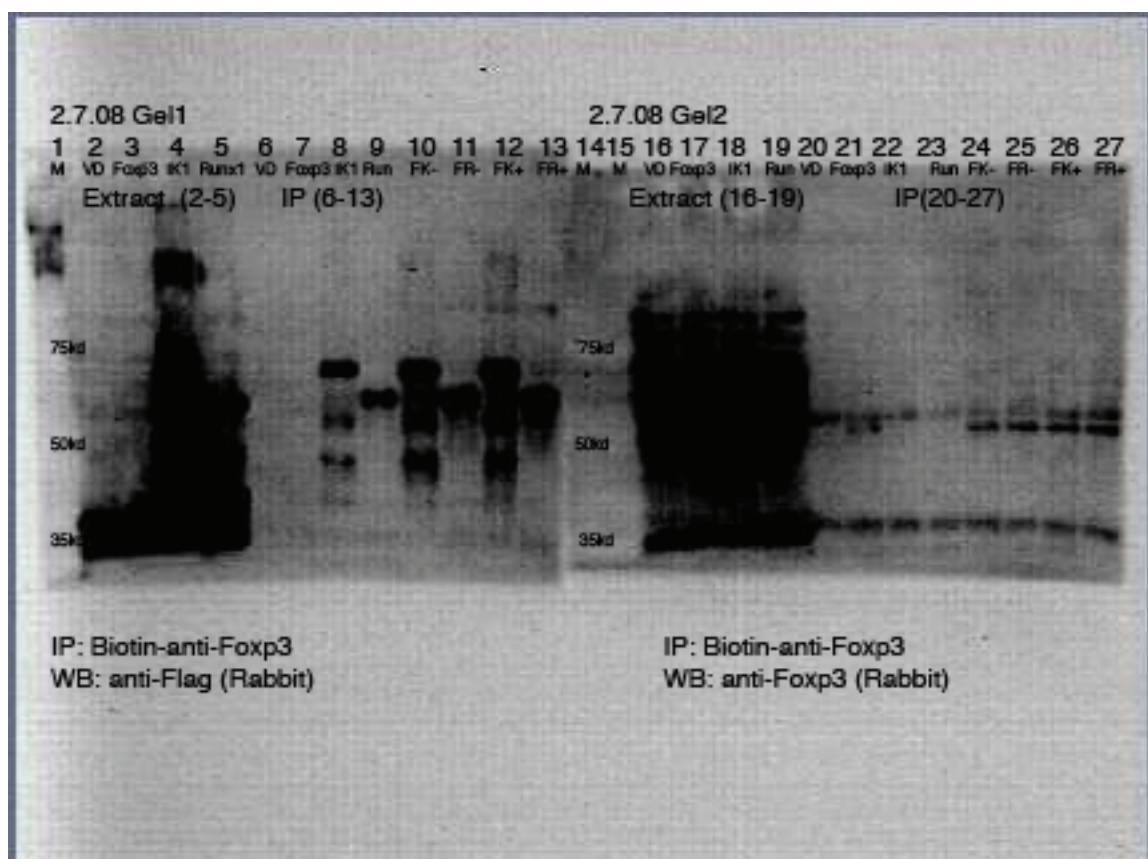

Supplement: Figure 6—source data 1. [file elife-91392-fig6-data1.pdf]
